# Shared genetic contribution to ischemic stroke and Alzheimer's disease

**DOI:** 10.1002/ana.24621

**Published:** 2016-03-30

**Authors:** Matthew Traylor, Poneh Adib‐Samii, Denise Harold, the Alzheimer's Disease Neuroimaging Initiative, The International Stroke Genetics Consortium (ISGC), UK Young Lacunar Stroke DNA resource, Martin Dichgans, Julie Williams, Cathryn M. Lewis, Hugh S. Markus, and the METASTROKE and International Genomics of Alzheimer's Project (IGAP) investigators, Myriam Fornage, Elizabeth G Holliday, Pankaj Sharma, Joshua C Bis, Bruce M Psaty, Sudha Seshadri, Mike A Nalls, William J Devan, Giorgio Boncoraglio, Rainer Malik, Braxton D Mitchell, Steven J Kittner, M Arfan Ikram, Robert Clarke, Jonathan Rosand, James F Meschia, Cathie Sudlow, Peter M Rothwell, Christopher Levi, Steve Bevan, Laura L Kilarski, Matthew Walters, Vincent Thijs, Agnieszka Slowik, Arne Lindgren, Paul I W de Bakker, Jean‐Charles Lambert, Carla A Ibrahim‐Verbaas, Denise Harold, Adam C Naj, Rebecca Sims, Céline Bellenguez, Gyungah Jun, Anita L DeStefano, Joshua C Bis, Gary W Beecham, Benjamin Grenier‐Boley, Giancarlo Russo, Tricia A Thornton‐Wells, Nicola Jones, Albert V Smith, Vincent Chouraki, Charlene Thomas, M Arfan Ikram, Diana Zelenika, Badri N Vardarajan, Yoichiro Kamatani, Chiao‐Feng Lin, Amy Gerrish, Helena Schmidt, Brian Kunkle, Melanie L Dunstan, Agustin Ruiz, Marie‐Thçrèse Bihoreau, Seung‐Hoan Choi, Christiane Reitz, Florence Pasquier, Paul Hollingworth, Alfredo Ramirez, Olivier Hanon, Annette L Fitzpatrick, Joseph D Buxbaum, Dominique Campion, Paul K Crane, Clinton Baldwin, Tim Becker, Vilmundur Gudnason, Carlos Cruchaga, David Craig, Najaf Amin, Claudine Berr, Oscar L Lopez, Philip L De Jager, Vincent Deramecourt, Janet A Johnston, Denis Evans, Simon Lovestone, Luc Letenneur, Francisco J Morón, David C Rubinsztein, Gudny Eiriksdottir, Kristel Sleegers, Alison M Goate, Nathalie Fiçvet, Matthew J Huentelman, Michael Gill, Kristelle Brown, M Ilyas Kamboh, Lina Keller, Pascale Barberger‐Gateau, Bernadette McGuinness, Eric B Larson, Robert Green, Amanda J Myers, Carole Dufouil, Stephen Todd, David Wallon, Seth Love, Ekaterina Rogaeva, John Gallacher, Peter St George‐Hyslop, Jordi Clarimon, Alberto Lleo, Anthony Bayer, Debby W Tsuang, Lei Yu, Magda Tsolaki, Paola Bossù, Gianfranco Spalletta, Petroula Proitsi, John Collinge, Sandro Sorbi, Florentino Sanchez‐Garcia, Nick C Fox, John Hardy, Maria Candida Deniz Naranjo, Paolo Bosco, Robert Clarke, Carol Brayne, Daniela Galimberti, Michelangelo Mancuso, Fiona Matthews, European Alzheimer's Disease Initiative (EADI), Genetic and Environmental Risk in Alzheimer's Disease (GERAD), Alzheimer's Disease Genetic Consortium (ADGC), Cohorts for Heart and Aging Research in Genomic Epidemiology (CHARGE), Susanne Moebus, Patrizia Mecocci, Maria Del Zompo, Wolfgang Maier, Harald Hampel, Alberto Pilotto, Maria Bullido, Francesco Panza, Paolo Caffarra, Benedetta Nacmias, John R Gilbert, Manuel Mayhaus, Lars Lannfelt, Hakon Hakonarson, Sabrina Pichler, Minerva M Carrasquillo, Martin Ingelsson, Duane Beekly, Victoria Alvarez, Fanggeng Zou, Otto Valladares, Steven G Younkin, Eliecer Coto, Kara L Hamilton‐Nelson, Wei Gu, Cristina Razquin, Pau Pastor, Ignacio Mateo, Michael J Owen, Kelley M Faber, Palmi V Jonsson, Onofre Combarros, Michael C O'Donovan, Laura B Cantwell, Hilkka Soininen, Deborah Blacker, Simon Mead, Thomas H Mosley, David A Bennett, Tamara B Harris, Laura Fratiglioni, Clive Holmes, Renee F A G de Bruijn, Peter Passmore, Thomas J Montine, Karolien Bettens, Jerome I Rotter, Alexis Brice, Kevin Morgan, Tatiana M Foroud, Walter A Kukull, Didier Hannequin, John F Powell, Michael A Nalls, Karen Ritchie, Kathryn L Lunetta, John S K Kauwe, Eric Boerwinkle, Matthias Riemenschneider, Mercè Boada, Mikko Hiltunen, Eden R Martin, Reinhold Schmidt, Dan Rujescu, Li‐San Wang, Jean‐François Dartigues, Richard Mayeux, Christophe Tzourio, Albert Hofman, Markus M Nöthen, Caroline Graff, Bruce M Psaty, Lesley Jones, Jonathan L Haines, Peter A Holmans, Mark Lathrop, Margaret A Pericak‐Vance, Lenore J Launer, Lindsay A Farrer, Cornelia M van Duijn, Christine Van Broeckhoven, Valentina Moskvina, Sudha Seshadri, Julie Williams, Gerard D Schellenberg, Philippe Amouyel

**Affiliations:** ^1^ Stroke Research Group, Department of Clinical NeurosciencesUniversity of CambridgeCambridgeUnited Kingdom; ^2^ Department of Medical & Molecular GeneticsKing's College LondonLondonUnited Kingdom; ^3^ Stroke and Dementia Research CenterSt George's University of LondonLondonUnited Kingdom; ^4^ School of BiotechnologyDublin City UniversityDublinIreland; ^5^ Institute for Stroke and Dementia ResearchKlinikum der Universität München, Ludwig‐Maximilians‐UniversitätMunichGermany; ^6^ Munich Cluster for Systems Neurology (SyNergy)MunichGermany; ^7^ Medical Research Council (MRC) Center for Neuropsychiatric Genetics and Genomics, Department of Psychological Medicine and Neurology, School of MedicineCardiff UniversityCardiffUnited Kingdom; ^8^ Social, Genetic and Developmental Psychiatry Center, Institute of PsychiatryKing's College LondonLondonUnited Kingdom

## Abstract

**Objective:**

Increasing evidence suggests epidemiological and pathological links between Alzheimer's disease (AD) and ischemic stroke (IS). We investigated the evidence that shared genetic factors underpin the two diseases.

**Methods:**

Using genome‐wide association study (GWAS) data from METASTROKE + (15,916 IS cases and 68,826 controls) and the International Genomics of Alzheimer's Project (IGAP; 17,008 AD cases and 37,154 controls), we evaluated known associations with AD and IS. On the subset of data for which we could obtain compatible genotype‐level data (4,610 IS cases, 1,281 AD cases, and 14,320 controls), we estimated the genome‐wide genetic correlation (rG) between AD and IS, and the three subtypes (cardioembolic, small vessel, and large vessel), using genome‐wide single‐nucleotide polymorphism (SNP) data. We then performed a meta‐analysis and pathway analysis in the combined AD and small vessel stroke data sets to identify the SNPs and molecular pathways through which disease risk may be conferred.

**Results:**

We found evidence of a shared genetic contribution between AD and small vessel stroke (rG [standard error] = 0.37 [0.17]; *p* = 0.011). Conversely, there was no evidence to support shared genetic factors in AD and IS overall or with the other stroke subtypes. Of the known GWAS associations with IS or AD, none reached significance for association with the other trait (or stroke subtypes). A meta‐analysis of AD IGAP and METASTROKE + small vessel stroke GWAS data highlighted a region (ATP5H/KCTD2/ICT1) associated with both diseases (*p* = 1.8 × 10^−8^). A pathway analysis identified four associated pathways involving cholesterol transport and immune response.

**Interpretation:**

Our findings indicate shared genetic susceptibility to AD and small vessel stroke and highlight potential causal pathways and loci. Ann Neurol 2016;79:739–747

Taken together, ischemic stroke (IS) and Alzheimer's disease (AD) place an enormous burden on global health care. Both are among the leading causes of acquired disability worldwide,^1^, ^2^ and the lifetime risk of AD or stroke is as high as 1 in 2 for women and 1 in 3 for men.^3^ An estimated 24.3 million people were thought to have dementia in 2001, and this is expected to rise to 81.1 million by 2040.^2^, ^4^

Increasing evidence suggests epidemiological and pathological links between AD and IS. Population‐based studies have shown AD to be a risk factor for developing IS,^5^, ^6^ and vice versa,^7^ suggesting that shared pathological processes may be involved in both conditions. Other studies have indicated a synergistic relationship between cerebral infarction and AD, with the combination of both leading to an increased risk of cognitive decline and dementia. Studies have also shown that cerebrovascular events lead to more‐rapid cognitive decline in patients with AD.^8^ Postmortem studies have shown that individuals with cerebral infarcts as well as neuropathological AD had a markedly increased risk of dementia in life compared with those with AD pathology without infarcts.^9^, ^10^, ^11^

The predominant vascular lesion in AD is cerebral amyloid angiopathy (CAA),^12^, ^13^ and although CAA is more prevalent in IS than controls, it is not a common cause of IS.^14^ The predominant cerebral vascular pathologies in IS are large vessel atherosclerosis (large vessel disease; LVD)^15^, ^16^ and small vessel arteriosclerosis (small vessel disease; SVD).^17^ Both pathologies have been associated with AD. In particular, at least one third of AD cases have pathological evidence of moderate or severe SVD.^18^ AD and vascular pathologies are, however, temporally and anatomically distinct. This has led to the hypothesis that cerebrovascular disease may make the brain more susceptible to AD pathology, potentially by impairing clearance of amyloid.^19^ Alternatively, AD and IS may represent independent, but convergent, diseases that share some pathophysiological processes and therefore may be expected to share genetic risk factors.

Genome‐wide association studies (GWAS) have recently begun to identify the common genetic component of many diseases such as AD and IS. More than 20 variants have now been identified that contribute to AD,^20^, ^21^, ^22^ and in IS recent progress has resulted in the identification of a number of variants, the majority of which have been associated with specific stroke subtypes.^23^, ^24^, ^25^, ^26^, ^27^, ^28^ Indeed, recent evidence from GWAS indicates that a large proportion of risk in complex diseases such as AD and IS is attributable to the combined effects of a large number of common genetic variants,^29^, ^30^ each conferring only a small amount of disease risk.^31^, ^32^, ^33^ Recent studies estimate the proportion of variance explained by the genetic contribution from common variants to AD and IS to be approximately 24.0% and 18.0%, respectively. 29, 34

Genomic‐relatedness‐matrix restricted maximum likelihood (GREML) methods have been one of the key methodological advances that, in recent years, have improved understanding of the common genetic influence on and between complex traits. These approaches use distant relatedness between individuals to infer heritability and coheritability of complex traits and have been used to provide insights into and between many diseases.^35^, ^36^, ^37^ We performed a bivariate GREML analysis in two large GWAS cohorts consisting of AD and IS cases and controls to assess evidence of a shared genetic contribution to the two diseases. We initially sought to determine whether there was evidence of a shared genetic contribution to IS and AD and then sought to determine whether this association was particular to a specific subtype of IS. Finally, through a meta‐analysis and subsequent pathway analysis of the two data sets, we sought to identify the molecular pathways through which shared disease risk may be conferred.

## Participants and Methods

### Study Design and Participating Cohorts

Two different sets of cases and controls were used in two different parts of the analysis (Table 1). For the meta‐analysis and subsequent pathway analysis (analyses i, iii, and iv), we used data from METASTROKE + and the International Genomics of Alzheimer's Project (IGAP) as follows.

**Table 1:** Data Description

	Summary‐Level Analysis		GREML Analysis	
	Cases	Controls	Cases	Controls
Alzheimer's disease	17,008	37,154	1,281	14,320
All ischemic stroke	15,916	68,826	4,610	
Cardioembolic stroke	3,127	60,861	1,013	
Large vessel stroke	2,876	54,866	1,231	
Small vessel stroke	3,651	58,657	851	

GREML = genomic‐relatedness‐matrix restricted maximum likelihood.

The METASTROKE + data set consisted of IS cases and controls derived from three previously published populations: METASTROKE,^38^ WTCCC2‐Immunochip,^39^ and UK Young Lacunar Stroke DNA resource.^40^ All cohorts consisted of IS cases of European ancestry from Europe, North America, and Australia together with controls of matched ancestry. Where possible, stroke cases were subtyped according to the Trial of Org 10172 in Acute Stroke Treatment (TOAST) classification system.^41^ Information on clinical subtypes was available for 60.1% of cases. In two centers, the Heart Protection study (N cases/controls = 578/468) and Rotterdam (N cases/controls = 376/5396), TOAST subtyping was not performed. All populations were genotyped on standard platforms from Affymetrix (Affymetrix, San Diego, CA) or Illumina (Illumina, San Diego, CA) and imputed separately to reference data sets. The results of the association analyses from each group (METASTROKE, WTCCC2‐Immunochip, and DNA‐lacunar) were meta‐analyzed using a fixed‐effects inverse‐variance weighted model using METAL.^42^ Single‐nucleotide polymorphisms (SNPs) available in a sufficient number of stroke cases (>1,000) were included in the meta‐analysis.

The IGAP is a large, two‐stage study based upon GWAS on individuals of European ancestry. In stage 1, the IGAP used genotyped and imputed data on 7,055,881 SNPs to meta‐analyze four previously published GWAS data sets consisting of 17,008 AD cases and 37,154 controls (the European Alzheimer's disease Initiative [EADI], the Alzheimer Disease Genetics Consortium [ADGC], the Cohorts for Heart and Aging Research in Genomic Epidemiology consortium [CHARGE], and the Genetic and Environmental Risk in AD consortium [GERAD]). In stage 2, 11,632 SNPs were genotyped and tested for association in an independent set of 8,572 AD cases and 11,312 controls. Finally, a meta‐analysis was performed combining results from stages 1 and 2. In this analysis, only results from stage 1 were used. All AD cases met criteria for either possible, probable (NINCDS‐ADRDA, DSM‐IV), or definite (CERAD) AD.

For the bivariate GREML analyses (analysis ii), we obtained genotype level data consisting of AD cases and controls from LOAD‐CIDR,^43^ the Alzheimer's Disease Neuroimaging Initiative (ADNI), and eMERGE (Group Health) 44; IS cases and controls from the WTCCC2 ischemic stroke study,^23^ the Australian Stroke Genetic Collaborative, and Besta Stroke Register (Milano)^38^ and independent controls from RADIANT,^45^ People of the British Isles (POBI),^46^ and the Health ABC studies.^47^ All were genotyped on the Illumina 610k, 660k, 1M, or 1.2M arrays. We performed quality control independently on each data set, at the minimum removing SNPs genotyped in less than 97% of individuals, SNPs diverging from Hardy‐Weinberg equilibrium (p<1e‐6), and SNPs with minor allele frequency <1%. We then merged all individuals together and included only SNPs present in >99.9% of individuals, again removing SNPs diverging from Hardy‐Weinberg equilibrium (p<1e‐6). We performed ancestry‐informative principal components analysis with smartpca (EIGENSTRAT), 48 removing any individuals more than 6 standard deviations from the mean on any of the first five principal components over five iterations. The remaining individuals were used to calculate the first 30 principal components.

### Statistical Analyses

#### Analysis of GWAS Significant Loci

We first determined whether any of the loci considered significant in large meta‐analyses of IS and AD were significantly associated with risk of the other disease. In selecting IS associated SNPs, we considered the five loci (9p21, *HDAC9, PITX2, ZFHX3*, and *12q24.12*) associated with stroke in METASTROKE and in a recent publication.^38^, ^39^ We set a *p*‐value threshold of *p* = 0.01 to determine significance, using the Bonferroni method to correct for five independent tests. In selecting AD‐associated SNPs, we considered all independent loci associated with AD in the largest meta‐analysis to date, as well as any other loci which have been identified since that publication.^22^, ^49^, ^50^ We identified 22 such associations and set the corresponding Bonferroni‐corrected *p*‐value threshold at *p* = 0.0023. Where SNPs were not available in METASTROKE (six SNPs), we evaluated the available SNP that most strongly tagged the associated SNP.

#### Genetic Correlation Analysis

To estimate the genetic correlation between AD and IS, we performed a bivariate GREML analysis using the GCTA package (version 1.24.2).^51^, ^52^ The approach involves estimating the distant relatedness between all individuals based on common SNPs represented on GWAS arrays. Linear mixed models are then used to estimate the proportion of (co)heritability explained by the relatedness between individuals. We present our results as the estimated genetic correlation between the two diseases, rG, which is a value between –1 and 1 that can be interpreted as the degree of genetic effects shared by the two traits. Intuitively, rG would be positive when AD‐IS pairs are more related than IS‐control and AD‐control pairs and negative when AD‐IS pairs are less related then IS‐control and AD‐control pairs. We tested whether the correlation was significantly different from zero (rG≠0), interpreting a significant test for rG≠0 as evidence that genetic liability was shared between the two traits. In our analysis, we included the first 10 principal components in each analysis and removed distantly related individuals above a suggested threshold (>0.025). Estimates can be upwardly biased if related individuals remain in the sample^32^; this threshold is included to avoid this problem from arising. In addition, we evaluated the sensitivity of the results by repeating the analysis using a stricter minor allele frequency threshold (0.05), genotyping missingness threshold (99.95%), relatedness threshold (0.02), and a greater number of principal components (20) in the model. In all analyses, only autosomal SNPs were included.

#### Meta‐analysis of AD With Small Vessel Stroke

The results of the 17,008 AD cases and 37,154 controls from IGAP and the 3,652 SVD cases and 58,657 controls from METASTROKE + were meta‐analyzed using Stouffer's method, as implemented in METAL.^42^ Because our aim was to identify the genetic variants contributing to both disorders, we attributed equal weights to each of the diseases. After meta‐analysis, we extracted only those SNPs with no evidence of heterogeneity between the two data sets (*p* > 0.01), thereby removing SNPs that were associated with only one of the two traits. The remaining SNPs were then taken forward for further investigation, and all others were discarded. We used the Zaykin method to correct for inflation attributable to shared controls.^53^

#### Identification of Shared Disease Pathways

We then determined whether the meta‐analyzed results showed significant enrichment for annotated pathways and gene sets from the Gene Ontology (GO), BIOCARTA, REACTOME, and Kyoto Encyclopedia of Genes and Genomes (KEGG) libraries. We used the gene set enrichment analysis (GSEA) approach, as implemented in the MAGENTA software package,^54^ to determine the enrichment within the 1,488 gene sets that had more than 15 and less than 200 genes in our data set. A *p* value of significance for each gene set was then calculated, along with a false discovery rate (FDR) *q* value, estimated using the Benjamini‐Hochberg procedure.^55^ We used an FDR threshold of 0.05 to identify associated gene sets. We used a second pathway analysis approach (GSEA‐VEGAS) to verify any associated sets as follows. We calculated gene‐level association statistics for all genes using VEGAS^56^ and then calculated a gene‐set enrichment statistic for each of the pathways. We calculated an empirical *p* value by sampling random gene sets with the same number of genes as the tested pathway and compared GSEA‐enrichment statistics between the simulated and tested pathways.

#### ADNI

Data used in the bivariate GREML analyses were obtained from the ADNI database (adni.loni.usc.edu). The ADNI was launched in 2003 as a public‐private partnership, led by Principal Investigator Michael W. Weiner, MD. The primary goal of ADNI has been to test whether serial magnetic resonance imaging, positron emission tomography, other biological markers, and clinical and neuropsychological assessment can be combined to measure the progression of mild cognitive impairment and early AD. For up‐to‐date information, see www.adni-info.org.

## Results

### Analysis of GWAS Significant Loci

None of the SNPs associated with IS were associated with AD (*p* > 0.1). Similarly, none of the SNPs associated with AD passed Bonferroni correction for association with IS or any of the three subtypes. The strongest association was with rs11870474 [ATP5H/KCTD2] for small vessel stroke (*p* = 0.0042; Supplementary Table 1). Indeed, the odds ratio (OR) for association of this SNP with small vessel stroke was larger than for association with AD (SVD, 1.26 [1.08–1.48]; AD, 1.18 [1.08–1.30]). In addition, 17 of 22 SNPs had a concordant allelic effect direction between SVD and AD (two‐sided, *p* = 0.017), suggesting that although these individual SNPs did not show statistical evidence of association with SVD, the set may be enriched for SNPs with true, but small, effects on SVD. Conversely, no excess in sharing of direction of effect was identified for all IS, or for the cardioembolic (CE) or LVD subtypes.

#### Genetic Correlation Analysis

**Figure 1:**
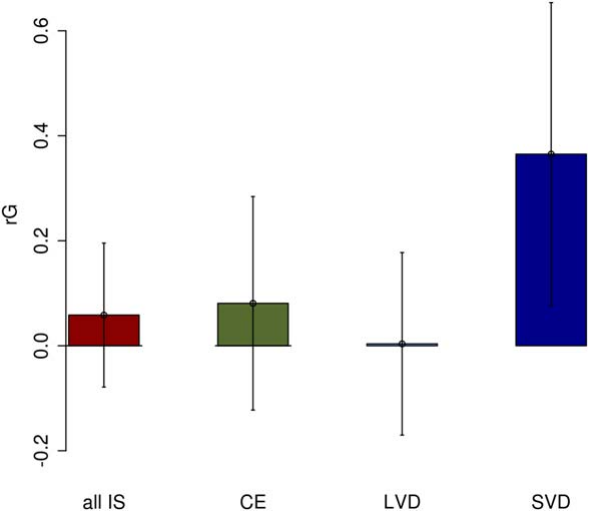
Genetic correlation between ischemic stroke (and ischemic stroke subtypes) and Alzheimer's disease showing estimate and 95% CI. CE = cardioembolic; CI = confidence interval; IS = ischemic stroke; LVD = large vessel disease; rG = genome‐wide genetic correlation; SVD = small vessel disease. [Color figure can be viewed in the online issue, which is available at www.annalsofneurology.org.]

A total of 278,233 SNPs and 22,208 individuals were used to construct the genetic relationship matrix used in the analysis. Univariate estimates of AD and IS heritability were in line with previous published estimates. There was a significant correlation between the genetic contribution underpinning SVD and AD (rG [standard error; SE] = 0.37 [0.17]; *p* = 0.011; Fig 1). Conversely, there was no evidence to support shared genetic factors underpinning AD and all IS (rG [SE] = 0.06 [0.08]; *p* = 0.24) or any of the other stroke subtypes: CE (rG [SE] = 0.08 [0.12]; *p* = 0.25) and LVD (rG [SE] = 0.00 [0.11]; *p* = 0.49). The association between SVD and AD was not affected by changes in minor allele frequency threshold (0.05), genotyping missingness threshold (99.95%), relatedness threshold (0.02), and number of principal components (20) in the model; all estimates of rG were between 0.35 and 0.37, and *p* values for rG>0 were all ≤ 0.026.

#### Meta‐analysis of AD With Small Vessel Stroke

**Figure 2:**
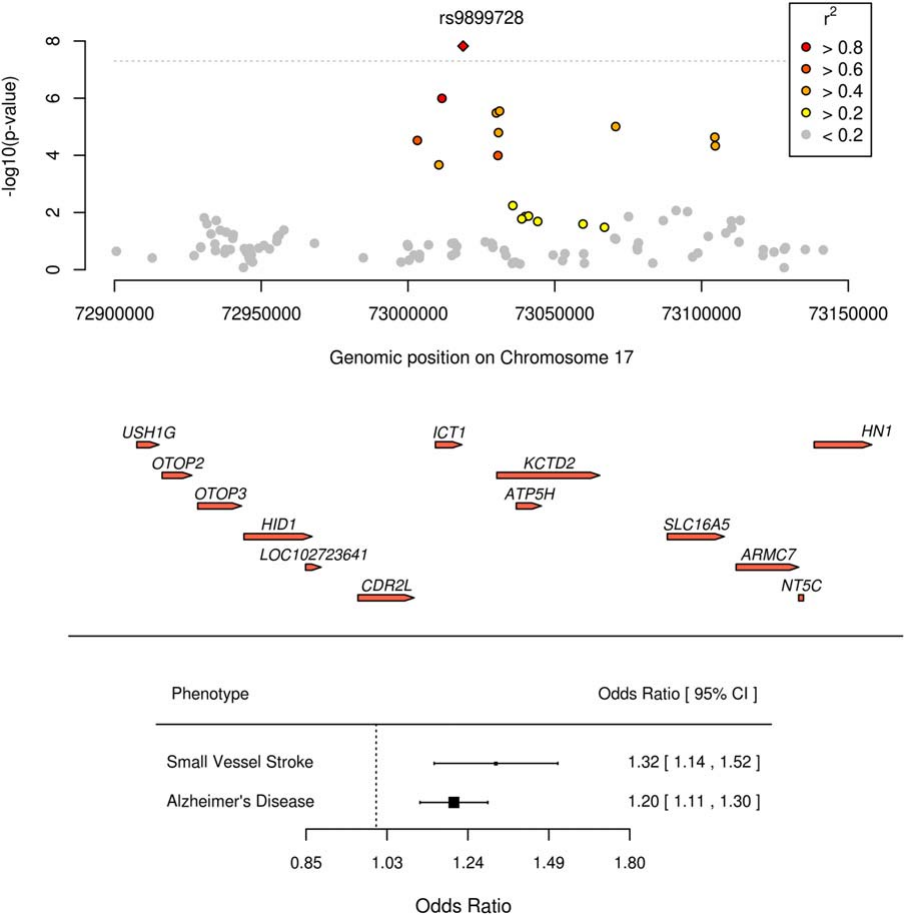
Association of ATP5H/KCTD2 locus with AD and SVD by genomic position and forest plot showing effect of risk SNP on SVD and AD with 95% CIs. AD = Alzheimer's disease; CI = confidence interval; SNP = single‐nucleotide polymorphism; SVD = small vessel disease. [Color figure can be viewed in the online issue, which is available at www.annalsofneurology.org.]

Having established that susceptibility to AD appears to be shared only with SVD, we performed a meta‐analysis of currently available GWAS data in SVD (METASTROKE+) and AD (IGAP). One region, near to *ATP5H*/*KCTD2*, was associated with AD and SVD at genome‐wide significance (p = 1.8 × 10^−8^; Fig 2). Variants in this region have been associated at genome‐wide significance with AD in a recent study,^49^ and this is the same region, albeit a different SNP in partial LD (r^2^=0.60), that produced the strongest association in section *i* above. This particular SNP, rs9899728, showed a larger effect on SVD (OR [95% confidence interval {CI}] = 1.32 (1.14–1.52); *p* = 0.00014) than AD (OR [95% CI] = 1.20 [1.11–1.30]; *p* = 7.1 × 10^−6^) and falls between two genes: Immature Colon Carcinoma Transcript 1 (*ICT1*) and adenosine triphosphate (ATP) synthase, H + transporting, mitochondrial Fo complex, subunit (*ATP5H*).

#### Identification of Disease Pathways Shared Between AD and SVD

The MAGENTA pathway analysis identified four significant pathways using the significance criteria of FDR *q* value < 0.05 (Table 2). Three of these involve lipid transport (GO/phospholipid efflux, GO/cholesterol efflux, and GO/reverse cholesterol transport), whereas one involves immune response (GO/negative regulation of nuclear factor kappa B [NF‐κB] transcription factor activity). When validating each significant pathway using an alternative method (GSEA‐VEGAS), all pathways were associated at *p* ≤ 0.011, whereas three were associated at *p* ≤ 0.0015.

**Table 2:** Significant Pathways Associated With SVD and AD From MAGENTA Analysis

		MAGENTA Analysis		GSEA‐VEGAS Validation
Gene set	No. of Genes in Set	*p*	FDR q Value	*p*
GO/phospholipid efflux	10	1.1 × 10^−5^	4.0 × 10^−4^	0.0015
GO/cholesterol efflux	20	1.0 × 10^−4^	1.2 × 10^−2^	0.0017
GO/reverse cholesterol transport	16	3.0 × 10^−4^	3.3 × 10^−2^	0.0018
GO/negative regulation of NF‐κB transcription factor activity	15	2.0 × 10^−4^	3.5 × 10^−2^	0.011

AD = Alzheimer's disease; FDR = false discovery rate; GO = gene ontology; GSEA = gene‐set enrichment analysis; NF‐κB = nuclear factor kappa B; SVD = small vessel disease; VEGAS = versatile gene‐based association statistic method.

## Discussion

Our results support the hypothesis that shared pathophysiological processes underlie both IS and AD, but suggest that this shared association is only with SVD and not with the other stroke subtypes. When investigating the genetic correlation between AD and IS, we found evidence that the cumulative effect of SNPs each individually conferring less risk, and therefore not reaching GWAS significance for stroke, are shared between AD and SVD, with no evidence to support association with any other stroke subtype.

Having established that AD and SVD share a substantial proportion of genetic susceptibility, we then sought to identify the genetic variants and shared molecular pathways through which AD and SVD disease risk is conferred. Our meta‐analysis highlighted one particular region on chromosome 17 in a region encompassing three genes; *ICT1*/*KCTD2*/*ATP5H*, which has previously been implicated in AD.^49^ In addition, we found evidence of association with four pathways. Three of these involve lipid transport, whereas the other involves immune response.

In addition, we used data from large GWASs in IS and AD to determine whether GWAS significant loci previously identified in AD are associated with stroke and whether known stroke loci associated with AD. We could find no significant associations, indicating that the contribution of specific associations identified thus far do not contribute a considerable proportion of genetic risk to the reciprocal disease.

The relationship between AD and IS is not fully understood. Vascular risk factors have been associated with AD,^57^ and a variety of cerebrovascular pathologies including IS have been reported to be more frequent in AD cases than controls.^15^, ^16^, ^17^ However, whether IS and AD pathologies are merely additive, or whether IS pathology directly promotes the development and progression of AD pathology, is debated.^57^ Possible mechanisms for the latter include cerebral hypoperfusion induced by cerebrovascular disease initiating or accelerating the neurodegeneration cascade, causing amyloid deposition and synaptic and neuronal dysfunction.^57^ In addition, whether there are shared pathological processes contributing to both IS and AD is uncertain. Our results offer new insights into this relationship. Previous studies have found that lacunar stroke, the hallmark of SVD, is associated with worse cognition in patients with AD pathology,^10^ although the relationship is still debated.^57^ A number of studies have reported that large artery atherosclerosis, in the carotid and intracranial arteries, is associated with AD risk.^15^, ^16^, ^58^ Our results do not support this and instead suggest that the most important cerebrovascular pathology mediating the relationship between IS and AD is SVD.

Our study has limitations. Cases from both IGAP and METASTROKE + are drawn from a number of international centers. We therefore cannot rule out the effect of differences in screening techniques and other diagnostic biases on our study. The relationship between IS and AD could be mediated, at least in part, by shared cardiovascular risk factors. We did not have risk factor data on all cases and controls to allow this to be adjusted for. However, if the association was driven by risk factors, we might expect this to affect all subtypes of stroke and would therefore not explain why our association is specific to small vessel stroke. When evaluating the *APOE ε4* allele, we used SNPs from HapMap II populations which are in partial linkage disequilibrium with the true variant. Without genotyping the causal allele, we cannot completely rule out an association, although if a strong association existed, we would expect it to be identified in our analysis. The diagnosis of AD was made using either established clinical criteria or pathophysiological confirmation; however, pathological studies having shown coexistent vascular pathology are not uncommon, and this could account for part of the shared risk. One other concern in analyses such as this, which include a large number of variables and individuals, is that subtle biases can influence the results. We took great care to only include samples that were genotyped on similar arrays from Illumina and directly assessed relatedness between individuals based on genotypes. In addition, our results were not sensitive to changes in parameters used, such as minor allele and relatedness thresholds.

In summary, our results demonstrate that a proportion of the common genetic susceptibility to AD is shared with SVD, but not with any other stroke subtypes. Our results provide a baseline for further study into the complex relationship between AD and SVD through genome‐wide analyses.

## Author Contributions

M.T., P.A.‐S, C.M.L., J.W., M.D., and H.S.M. designed the experiment. M.T., P.A.‐S., and D.H. carried out the statistical analysis. M.T., P.A.‐S., and H.S.M. wrote the first draft of the manuscript. M.T drew the figures. All authors reviewed and commented on the manuscript. METASTROKE and IGAP investigators who were responsible for collecting, phenotyping, or analysing the original cohorts are indicated in the attached supplemental file. M.T. and P.A.‐S. contributed equally to this work.

## Potential Conflicts of Interest

Nothing to report.

## Supporting information

Additional supporting information can be found in the online version of this article.

Supporting Information
