# Supplementary material for: Shared genetic contribution to ischemic stroke and Alzheimer's disease
Source: Ann Neurol. 2016 Mar 30;79(5):739–47. doi: 10.1002/ana.24621 (PMC4864940; doi:10.1002/ana.24621)
Supplement: Supplementary file 1 — Supporting Information [file ANA-79-739-s001.docx]

**Supplementary Table I – Association of Alzheimer’s disease associated SNPs with ischaemic stroke and its subtypes**

| SNP [Gene] | RA | OA | AD, OR(95% CI) | SVD, OR(95% CI); p | LVD, OR(95% CI); p | CE, OR(95% CI); p | IS, OR(95% CI); p |
| --- | --- | --- | --- | --- | --- | --- | --- |
| rs6857 [APOE] | T | C | 3.19 (3.05–3.34) | 1.00 (0.92-1.09); 0.94 | 0.98 (0.89-1.08); 0.74 | 0.95 (0.87-1.04); 0.27 | 0.96 (0.92-1.01); 0.09 |
| rs10792832 [PICALM] | A | G | 0.88 (0.85-0.91) | 0.98 (0.92-1.04); 0.44 | 0.97 (0.90-1.03); 0.30 | 0.96 (0.90-1.02); 0.16 | 0.98 (0.95-1.01); 0.19 |
| rs2847666 [MS4A6A] | A | G | 1.10 (1.07–1.14) | 1.01 (0.94-1.07); 0.81 | 0.96 (0.89-1.03); 0.24 | 0.99 (0.92-1.06); 0.73 | 1.00 (0.97-1.04); 1.00 |
| rs10948363 [CD2AP] | A | G | 0.91 (0.88-0.94) | 0.95 (0.88-1.01); 0.12 | 1.02 (0.94-1.10); 0.60 | 1.01 (0.94-1.08); 0.81 | 1.01 (0.97-1.04); 0.72 |
| rs7568027 [INPP5D] | A | G | 0.94 0.91-0.97) | 1.03 (0.97-1.09); 0.31 | 1.03 (0.96-1.10); 0.46 | 1.02 (0.95-1.08); 0.65 | 1.01 (0.98-1.04); 0.54 |
| rs2722295 [NME8] | T | C | 0.93 0.90-0.97) | 0.99 (0.92-1.05); 0.67 | 1.00 (0.92-1.07); 0.91 | 1.02 (0.95-1.09); 0.62 | 1.00 (0.97-1.04); 0.84 |
| rs2322599 [PTK2B] | A | G | 1.10 (1.06-1.13) | 0.95 (0.89-1.01); 0.08 | 1.00 (0.94-1.07); 0.97 | 1.04 (0.98-1.11); 0.15 | 0.99 (0.96-1.02); 0.52 |
| rs3752246 [ABCA7] | C | G | 0.87 (0.84-0.91) | 0.96 (0.89-1.04); 0.34 | 0.95 (0.86-1.04); 0.28 | 0.94 (0.86-1.03); 0.18 | 0.99 (0.94-1.03); 0.52 |
| rs17125944 [FERMT2] | T | C | 0.88 (0.84-0.93) | 0.97 (0.88-1.08); 0.61 | 0.99 (0.89-1.11); 0.87 | 0.99 (0.89-1.10); 0.85 | 0.98 (0.93-1.03); 0.35 |
| rs11870474 [ATP5H*] | A | C | 1.18 (1.08-1.30) | 1.26 (1.08-1.48); 0.0042* | 0.90 (0.72-1.12); 0.35 | 1.08 (0.90-1.30); 0.39 | 0.98 (0.89-1.08); 0.69 |
| rs1476679 [ZCWPW1] | T | C | 1.08 (1.04-1.12) | 1.05 (0.98–1.12); 0.18 | 1.05 (0.98-1.14); 0.16 | 1.08 (1.00-1.15); 0.04 | 1.01 (0.98-1.05); 0.42 |
| rs11771145 [EPHA1] | A | G | 0.90 (0.87-0.93) | 1.04 (0.98-1.10); 0.24 | 1.04 (0.97-1.12); 0.27 | 1.08 (1.01-1.15); 0.03 | 1.02 (0.98-1.05); 0.32 |
| rs6656401 [CR1] | A | G | 1.17 (1.12-1.22) | 1.03 (0.96-1.11); 0.39 | 1.05 (0.97-1.12); 0.24 | 0.95 (0.88-1.02); 0.17 | 1.00 (0.96-1.04); 0.91 |
| rs10838726 [CELF1] | C | G | 0.93 (0.90-0.96) | 1.07 (1.01-1.14); 0.032 | 0.99 (0.92-1.06); 0.69 | 0.99 (0.93-1.06); 0.80 | 1.01 (0.98-1.05); 0.39 |
| rs10498633  [SLC24A4-RIN3] | T | G | 0.90 (0.87-0.94) | 0.98 (0.91-1.05); 0.59 | 1.00 (0.93-1.08); 0.96 | 0.99 (0.92-1.06); 0.77 | 1.00 (0.96-1.04); 0.95 |
| rs927174 [CASS4] | A | C | 1.13 (1.07-1.19) | 1.01 (0.90-1.12); 0.90 | 1.08 (0.96-1.21); 0.22 | 0.99 (0.89-1.10); 0.78 | 1.02 (0.97-1.08); 0.41 |
| rs111418223 [HLA] | A | C | 0.90 (0.87-0.93) | 0.92 (0.83-1.01); 0.081 | 0.98 (0.82-1.16); 0.81 | 1.13 (0.99-1.30); 0.07 | 1.03 (0.95-1.12); 0.45 |
| rs11218343 [SORL1] | T | C | 1.31 (1.21-1.42) | 1.02 (0.82-1.28); 0.83 | 0.83 (0.65-1.05); 0.12 | 0.81 (0.64-1.01); 0.06 | 0.95 (0.84-1.08); 0.46 |
| rs9331896 [CLU] | T | C | 1.16 (1.12-1.20) | 1.01 (0.95-1.08); 0.64 | 1.02 (0.95-1.09); 0.57 | 0.99 (0.94-1.06); 0.87 | 0.99 (0.96-1.02); 0.58 |
| rs190982 [MEF2C] | A | G | 1.08 (1.05-1.12) | 0.98 (0.92-1.05); 0.57 | 1.03 (0.96-1.11); 0.44 | 1.04 (0.98-1.12); 0.21 | 1.02 (0.99-1.06); 0.20 |
| rs6733839 [BIN1] | T | C | 1.21 (1.17-1.25) | 1.03 (0.90-1.18); 0.63 | NA | NA | NA |
| rs74615166 [TRIP4*] | T | C | 1.40 (1.22-1.61) | 1.28 (0.81-2.00); 0.29 | NA | NA | NA |
| Shared direction  (p-value) |  |  |  | 17 of 22 (0.017) | 8 of 20 (0.50) | 11 of 20 (0.82) | 10 of 20 (1.00) |

AD, Alzheimer’s disease; SVD, small vessel disease; LVD, large vessel disease; CE, cardioembolic stroke; IS, all ischaemic stroke; OR, odds ratio; CI, confidence interval; p-value for evidence of shared direction of effect derived from a two-tailed binomial test. RA, reference allele; OA, other allele.
